# Physical exercise and academic performance among students in the medical field at Jordanian Universities

**DOI:** 10.1371/journal.pone.0341310

**Published:** 2026-01-22

**Authors:** Ahlam J. Alhemedi, Sawsan Abuhammad, Thekraiat Majed A.L. Quran, Omar Khasawneh, Motaz Al-Yafeai, Mohammed Al-Wazeer

**Affiliations:** 1 Department of Public Health and Family Medicine, Faculty of Medicine, Jordan University of Science and Technology, Irbid, Jordan; 2 Department of Maternal Child Health and Midwifery, Faculty of Nursing, Jordan University of Science and Technology, Irbid, Jordan; 3 Department of Nursing, College of Health Sciences, University of Sharjah, Sharjah, United Arab Emirates; University of Petra (UOP), JORDAN

## Abstract

**Background:**

The literature emphasizes multiple factors that affect academic performance, including emotional and physical well-being, motivation, and physical exercise. University students, especially medical students, usually lack time and experience high levels of stress, leading to neglect of a healthy lifestyle. This study aimed to assess the association between physical activity patterns and academic performance (cumulative grade point average (cGPA)) among medical students at Jordanian universities.

**Methods:**

This is an online survey study that was conducted in Jordan between 28/12/2023 and 30/07/2025. A multivariate logistic regression analysis was performed to identify sociodemographic and physical activity items associated with academic performance, while sociodemographic characteristics were analyzed to predict the probability of engaging in physical activities among participating students.

**Results:**

A total of 1,209 university students participated in this study. Around 54.8% reported engaging in physical activity. Vigorous exercises such as running or football were practiced by 61.8%, and moderate activities such as brisk walking or swimming by 74.8%. Nearly half of the participants (45.9%) had been exercising for over a year, and 42.7% exercised more than 2.5 hours weekly. The main motivations for physical activity included maintaining health (39.9%) and stress relief (31.8%), while barriers included a lack of time (33.1%) and energy (26.9%). Students aged 22–24 were significantly less likely to have cGPA > 3.5 compared to those aged 18–21 (adjusted odds ratio (aOR) = 0.6, 95% confidence interval (CI): 0.4–0.9; p = 0.021). Additionally, non-Jordanian students had a lower likelihood of achieving a cGPA > 3.5 compared to Jordanian students (aOR = 0.3; 95% CI: 0.2–0.5; p < 0.001).

**Conclusion:**

In this study sample, medical students exercise moderately. Health improvement and stress relief were the main motivators, whereas a lack of time and energy were barriers. Significant cGPA variances were seen according to age, university, and nationality, revealing sociocultural and institutional impacts within this study population. Although physical activity was not significantly associated with academic performance, females showed lower odds of participating in physical activity, highlighting the importance of promoting physical activity among women to improve overall student health and well-being.

## Introduction

The academic performance of medical students is the principal objective of medical education. High educational achievement is linked with better career opportunities and professional success [1–3]. In contrast, academic failure is associated with several difficulties, including low learning and education quality, as well as an increased unemployment rate [4]. As such, determining the factors that influence academic performance is important [5]. Literature emphasizes multiple factors that affect academic performance [6], including emotional and physical well-being, motivation [7], and physical exercise [8–10]. Research has shown that physical exercise can enhance academic performance by improving cognitive functions, such as memory, concentration, perception, and decision-making [8–11]. Additionally, it is also associated with a better quality of life, enhanced mental health, and is a fundamental factor in preventing considerable diseases [12,13]. It is important to note, exercise’s impact on academic performance is affected by frequency, duration, and type [14,15].

University students, especially medical students [16–20], usually lack time and experience high levels of stress, leading them to neglect a healthy lifestyle, including not participating in regular physical activity [21]. Moreover, medical students experience multiple mental health problems, including stress, depression, and anxiety [22–27]. In addition, sedentary behavior is linked to an increased risk of chronic diseases (depression, obesity, cancer, type 2 diabetes, and heart disease) and a higher mortality rate [28]. Thus, a healthy lifestyle (sleep, diet, and exercise) is critical to preserving medical students’ well-being [22]. A previous study showed that medical students and physicians who follow a healthy lifestyle are more effective in discussing healthy lifestyle choices with patients, and patients trust their recommendations more [29].

Physical activity offers multiple benefits for medical students, including improving their quality of life and academic performance [30], as well as decreasing stress, burnout, and depression [17,31–34]. Research has also reported that academic performance and engagement in physical activity are influenced by students’ body mass index, age, and gender [35,36]. Understanding the association between academic performance and physical exercise is complex and highly significant. The bulk of earlier investigations demonstrated a positive association between academic performance and levels of physical activity [37–42]; however, few investigations revealed a negative association [43,44] or a non-significant association [30,45]. Despite the global evidence linking physical activity and academic performance, few studies have explored this association in the Middle East or amongst Jordanian students, where sociocultural norms and academic stress may affect exercise habits. Understanding this relationship among Jordanian medical students is essential for the development of academic programs. This study aimed to assess the association between physical activity and academic performance among medical students at Jordanian universities. We hypothesized that students who engage more frequently in physical exercise would demonstrate higher academic achievement.

## Methods

### Study design

This is an online survey study that was conducted in Jordan between 28/12/2023 and 30/07/2025. Data collection and recruitment occurred across three main time windows to increase coverage of medical students during different academic periods. The first wave was conducted between December 2023 and February 2024, which was the mid-academic semester, the second wave was conducted between May and July 2024, which was the final exam period, and the last wave was conducted between March and the end of July 2025 to include the following semester and summer semester students. Recruitment across these time windows was intended to include students from different phases to reduce the potential risk of response bias due to exams.

### Study population and sampling strategy

The study population consisted of active university students currently enrolled in medical, dental, or PharmD programs in Jordan. The inclusion criteria were students aged 18 years or older who were actively attending classes in a medical-related field. Exclusion criteria included students who were not currently enrolled, were on leave of absence, or did not agree to participate. Students with chronic illness, disabilities or other conditions were excluded from the study as these factors could independently influence their physical activity levels and academic performance. No subgroup analyses were performed due to the small number of participants.

### Survey administration

We employed the convenience sampling technique to recruit study participants who met the inclusion criteria. The survey was distributed online via social media platforms, such as Facebook and WhatsApp. The invitation letter clearly mentioned the study purpose, inclusion criteria, and that students participated voluntarily. To facilitate anonymity, no identifying personal information, such as name, email, or student identification document (ID), was collected, and responses were recorded anonymously. Participants were informed that completing the survey implied consent to participate in the study, which aligned with ethical guidelines for online surveys.

### Questionnaire tool

We developed an anonymous questionnaire to examine the association between physical activity and academic performance among medical students in Jordan based on a previous literature review. The questionnaire tool comprised two sections. The first section consisted of seven items to examine the sociodemographic and academic characteristics of the study participants (gender, age, university name, nationality, speciality, cumulative grade point average (cGPA), and cGPA in symbols), which were assessed using multiple-choice and categorical response options. The second section included 10 items on physical activity patterns, frequency, duration, types of exercise, motivations and barriers, using a combination of multiple-choice and yes/no format questions. The sports injury question referred to lifetime history (ever having had a sports-related injury).

The initial survey instrument was pilot tested on a sample of 25 participants. Piloting the instrument confirmed the clarity of the questionnaire tool with no suggested modifications.

### Ethical approval

Ethical approval for this study was obtained from the Institutional Review Board at Jordan University of Science and Technology, Irbid, Jordan (No: 807/2023). Participants were informed that completing the questionnaire was considered informed consent.

### Data analysis

Data was analyzed using IBM Statistical Package for Social Science (SPSS) Statistics version 31. Descriptive statistics such as frequency and percentage were utilized to summarize categorical data such as demographic characteristics and other physical activity items. The primary outcome was high academic performance, defined as cGPA > 3.5. This cutoff point was determined based on the university’s grading system, with greater than 3.5 representing a distinguished cGPA. A multivariate logistic regression was conducted to examine factors associated with high academic performance, including sociodemographic characteristics, such as age, gender, nationality, and university, as well as physical activity data, such as frequency and duration of activity. Physical activity was treated as a key predictor alongside demographic covariates. Adjusted odds ratio (aOR) with 95% confidence interval (CI) and p-values were reported. All statistical analyses were two-tailed, conducted at a significance level of p < 0.05.

## Results

A total of 1,209 university students participated in the study. 537 (44.4%) were males and 672 (55.6%) were females. Most participants were aged 22–24 years, totaling 818 students (67.7%). Most students were from the Jordan University of Science and Technology (n = 1,012, 83.7%). The majority were Jordanian nationals (n = 1,008, 83.4%), and most were enrolled in the medicine program (n = 1,078, 89.2%). Further details about sociodemographic and academic characteristics are provided in Table 1.

**Table 1:** Sociodemographic and academic characteristics.

Sociodemographic and academic characteristics		N	%
Gender	Male	537	44.4%
	Female	672	55.6%
Age (years)	18-21	274	22.7%
	22-24	818	67.7%
	25 and older	117	9.7%
University	Jordan University of Science and Technology	1012	83.7%
	The University of Jordan	48	4.0%
	Yarmouk University	37	3.1%
	The Hashemite University	81	6.7%
	Mutah University	31	2.6%
Nationality	Jordanian	1008	83.4%
	Other	201	16.6%
Specialty	Medicine	1078	89.2%
	Dentistry	67	5.5%
	PharmD	64	5.3%
Cumulative GPA	4-4.2	105	8.7%
	3.5-3.99	604	50.0%
	3-3.49	393	32.5%
	<3	107	8.9%
Cumulative GPA in symbol	A	610	50.5%
	B	480	39.7%
	C	119	9.8%

GPA: Grade point average.

Of 1,209 university students, 663 (54.8%) reported engaging in physical activity. 410 (61.8%) participants did vigorous exercises, such as running or football, and 496 (74.8%) reported doing moderate activities such as brisk walking or swimming. Nearly half (45.9%) had been exercising for over a year, and 283 (42.7%) exercised more than 2.5 hours weekly. The main motivations for doing exercise included maintaining health (39.9%) and stress relief (31.8%), while barriers included a lack of time (33.1%) and energy (26.9%). See Table 2 for more detail.

**Table 2:** Physical activity patterns, motivations and barriers among university students.

Physical activity patterns, motivations and barriers		N	%
Do you do any type of physical exercise?	Yes	663	54.8%
**Do you do any vigorous-intensity sports, fitness or recreational (leisure) activities that cause large increases in breathing or heart rate for at least 10 minutes continuously?**			
Running or football	Yes	410	61.8%
Brisk walking, cycling, swimming, volleyball	Yes	496	74.8%
For how long have you been doing regular physical exercise?	< 6 months	238	35.9%
	6 months – 1 year	121	18.3%
	> 1 year	304	45.9%
Approximately how long do you exercise each week?	0-2.5 hours (0–150 minutes) weekly	380	57.3%
	> 2.5 hours (more than 150 minutes) week	283	42.7%
**What types of exercises do you usually do?**			
Brisk Walking	Yes	456	68.8%
Running	Yes	353	53.2%
Swimming	Yes	151	22.8%
Aerobic exercises	Yes	298	44.9%
Football	Yes	228	34.4%
Heavy lifting	Yes	310	46.8%
How many days per week do you exercise?	Daily	159	24.0%
	Every other day	298	44.9%
	Twice a week	129	19.5%
	Once a week	77	11.6%
Where do you usually exercise?	Home	219	33.0%
	Gym	277	41.8%
	Outdoor	167	25.2%
**What is your motivation for doing physical exercises?**			
Maintaining health	Yes	482	39.9%
Leisure	Yes	129	10.7%
Stress relief and relaxation	Yes	385	31.8%
Physical appearance &body building	Yes	372	30.8%
**What are the main reasons preventing you from doing physical exercises?**			
Lack of time	Yes	400	33.1%
Lack of energy	Yes	325	26.9%
Lack of resources (money, places, transportation)	Yes	197	16.3%
Lack of motivation	Yes	314	26.0%
Social influences	Yes	22	1.8%
Fear of injury	Yes	22	1.8%
Having a chronic illness	Yes	25	2.1%
I think it is not important	Yes	15	1.2%
Have you ever had a sports injury?	Yes	344	28.5%
**What type of injury have you had?**			
Fracture	Yes	95	7.9%
Dislocation	Yes	51	4.2%
Sprain (tear of a ligament)	Yes	142	11.7%
Strain (tear in tendon or muscle)	Yes	108	8.9%
Tendinitis	Yes	51	4.2%
Bursitis	Yes	31	2.6%

Students aged 22–24 were significantly less likely to have cGPA > 3.5 compared to those aged 18–21 (aOR = 0.6, 95% CI: 0.4–0.9; *p* = 0.021). Compared to Jordan University of Science and Technology students, those from The University of Jordan (aOR= 0.3, 95% CI: 0.1–0.8; *p* = 0.018) and The Hashemite University (aOR = 0.2; 95% CI: 0.1–0.5; *p* < 0.001) were significantly less likely to have a higher cGPA. Additionally, non-Jordanian students were less likely to achieve a cGPA > 3.5 compared to Jordanian students (aOR = 0.3; 95% CI: 0.2–0.5; *p* < 0.001). See Table 3.

**Table 3:** Factors associated with higher academic performance.

Factors associated with higher academic performance		aOR (95% CI)	P value
Gender	Male	Reference	
	Female	1.2 (0.8–1.8)	0.407
Age (years)	18-21	Reference	
	22-24	0.6 (0.4–0.9)	0.021
	25 and older	0.5 (0.3–1.1)	0.084
University	Jordan University of Science and Technology	Reference	
	The University of Jordan	0.3 (0.1–0.8)	0.018
	Yarmouk University	1.0 (0.4–2.6)	0.991
	The Hashemite University	0.2 (0.1–0.5)	< 0.001
	Mutah University	0.5 (0.2–1.4)	0.182
Nationality	Jordanian	Reference	
	Others	0.3 (0.2–0.5)	< 0.001
Specialty	Medicine	Reference	
	Dentistry	1.2 (0.5–2.5)	0.697
	PharmD	0.9 (0.4–2.1)	0.805
Do you do any vigorous-intensity sports, fitness or recreational (leisure) activities that cause large increases in breathing or heart rate, such as running or football, for at least 10 minutes continuously?	No	Reference	
	Yes	0.9 (0.6–1.3)	0.428
Do you do any moderate-intensity sports, fitness or recreational (leisure) activities that cause a small increase in breathing or heart rate, such as brisk walking, cycling, swimming or volleyball, for at least 10 minutes continuously?	No	Reference	
	Yes	1.3 (0.8–1.9)	0.252
For how long have you been doing regular physical exercise?	< 6 months	Reference	
	6 months – 1 year	0.9 (0.5–1.4)	0.567
	> 1 year	1.3 (0.9–1.9)	0.235
Approximately how long do you exercise weekly?	0-2.5 hours (0–150 minutes) weekly	Reference	
	> 2.5 hours (more than 150 minutes) week	1.2 (0.8–1.8)	0.431
	if you do exercises for more than 150 minutes (>;2.5 hours) weekly; please write down approximately how many hours you exercise per week?	1.0 (1.0–1.0)	0.979
How many days per week do you exercise?	Daily	Reference	
	Every other day	1.1 (0.7–1.6)	0.793
	Twice weekly	1.2 (0.7–2.0)	0.534
	Once weekly	1.0 (0.5–1.8)	0.884
Where do you usually exercise?	Home	Reference	
	Gym	1.1 (0.7–1.8)	0.623
	Outdoor	1.2 (0.7–1.9)	0.504

aOR: adjusted odds ratio.

Female students were significantly less likely to engage in physical activity compared to males (aOR = 0.5; 95% CI: 0.4–0.6; p < 0.001). No other variables, including age, university, nationality, specialty, or cGPA, showed a statistically significant association with physical activity in this model. See Table 4.

**Table 4:** Factors associated with physical activity engagement.

Factors associated with physical activity engagement		aOR (95% CI)	P value
Gender	Male	Reference	
	Female	0.5 (0.4–0.6)	< 0.001
Age (years)	18-21	Reference	
	22-24	1.2 (0.9–1.6)	0.187
	25 and older	1.0 (0.6–1.6)	0.976
University	Jordan University of Science and Technology	Reference	
	The University of Jordan	0.9 (0.5–1.8)	0.856
	Yarmouk University	1.6 (0.8–3.2)	0.189
	The Hashemite University	1.4 (0.9–2.4)	0.150
	Mutah University	0.7 (0.3–1.5)	0.336
Nationality	Jordanian	Reference	
	Other	1.0 (0.7–1.4)	0.920
Specialty	Medicine	Reference	
	Dentistry	1.2 (0.7–2.1)	0.437
	PharmD	1.0 (0.6–1.7)	0.920
cGPA	<3.5	Reference	
	≥ 3.5	1.3 (1.0–1.6)	0.071

aOR: adjusted odds ratio; cGPA: cumulative grade point average.

## Discussion

The results of this study indicate that around 54.8% of medical students engage in physical activity. Vigorous exercises, such as running or football, were performed by 61.8%, and moderate activities such as brisk walking or swimming, were performed by 74.8%. Females were less likely to do physical exercise compared to males. Age group and university of study were factors associated with variation in academic performance.

Medical students in Jordan face unique institutional and cultural constraints that may contribute to lower physical activity levels and academic performance differences. In our results, only 54.8% reported exercising; this low percentage is mainly due to long study hours and an intense curriculum. Our results also showed that females were less likely to engage in physical activity, likely due to restrictions in the Middle East [46,47]. Students aged 22–24 and non-Jordanians were less likely to achieve high academic performance (cGPA > 3.5), possibly due to a higher academic load and challenges related to adapting to a different environment.

In comparison, prior studies among medical students found that about 81% of the students in Poland [48], 66% in Brazil [49], 62% in India [50,51], 56% in Sudan [52], 53% in Ireland [53], 47% in Saudi Arabia [42], and 32% in Lithuania [54] engage some form of regular physical activity. The observed differences might be attributed to factors related to colleges, including busy curricula, sports facilities, and physical activity programs [55]. For instance, an earlier investigation revealed an association between regular physical activity among medical students, the availability of sports facilities, and the college schedule [48]. Moreover, factors such as students’ gender and knowledge also affect the likelihood of engaging in physical activity. The bulk of prior research has highlighted that males tend to engage in physical activity more than females [56], which aligns with our findings. Indeed, regular physical activity is linked with many health, economic, academic, and social benefits [50,57]. Therefore, there is a need to increase participation in regular physical activity among medical students in Jordan. Improving medical students’ knowledge about the health benefits of physical activity will be beneficial [48]. In addition, it is essential to take into account specific college and student-related factors when developing any interventions aimed at increasing students’ physical activity.

This study showed that maintaining health (39.9%) and relieving stress (31.8%) were the primary motivations for engaging in physical activity, while a lack of time (33.1%) and energy (26.9%) were the most significant barriers. These findings align with several prior studies among medical students in India, Poland, the Western Balkans, and the United States, which report that improving stamina [51] and well-being [58,59], enhancing physical appearance [51,58,59], promoting mental health [60], reducing stress [59], and losing weight [51] were the most commonly reported motivations for doing physical exercise. Consistent with our findings, the leading barriers identified in earlier investigations were a lack of time [51,58,60,61] and energy [22]. Faculty obligations [59] and excessive study load [52] were also among the most prevalent physical activity barriers among medical students. Understanding these motivations and barriers is crucial, as the literature documents the association between motivation and levels of physical activity [61]. Moreover, low levels of physical activity were linked to poor mental health and academic performance [62]. Consequently, addressing the identified barriers is critical. Research highlighted possible solutions to overcome these barriers including making students’ schedule more consistent providing specific exercise breaks [22].

Our study revealed that students aged 22–24 were significantly less likely to have a cGPA > 3.5 compared to those aged 18–21 (aOR = 0.6, 95% CI: 0.4–0.9; p = 0.021). Additionally, compared to Jordan University of Science and Technology students, those from The University of Jordan (aOR = 0.3, 95% CI: 0.1–0.8; p = 0.018) and The Hashemite University (aOR = 0.2; 95% CI: 0.1–0.5; p < 0.001) were significantly less likely to have a higher cGPA. Additionally, non-Jordanian students were less likely to achieve a cGPA > 3.5 compared to Jordanian students (aOR = 0.3; 95% CI: 0.2–0.5; p < 0.001). These findings match those observed in earlier studies. For example, a prior survey among medical students revealed a significant association between age and cGPA; the lower the students’ age, the higher their cGPA [63]. Similarly, several previous studies demonstrated significantly better academic performance among younger students than older ones [64–67]. These differences in academic performance may reflect family obligations, economic problems [64,68], stress [69,70], and obstacles to learning and adaptation [71–75] among older students. Thus, targeted academic interventions are necessary for older students.

The literature also supports the observation of a higher cGPA among Jordan University of Science and Technology students compared to other university students, and among Jordanian students compared to non-Jordanian students. Each university has a different admissions process, learning environment, and curriculum, and all these aspects are documented to influence students’ academic performance [76–82]. Consistent with this study, international students and medical students in the United Kingdom [83] and New Zealand [84] have poor academic performance as a result of mental health challenges and concerns about quality of life. Other studies among medical students have shown an association between mental health issues and low educational achievement [85] or low cGPA [62]. Therefore, it is imperative to identify any mental health problems among these students early [83] and manage them effectively. This may lead to improved academic performance among medical students. Other related factors identified in the literature include coping strategies, cultural factors, English language ability, marital status, gender, and age [70,85–89]. As such, it is necessary to consider these factors when developing interventions to enhance medical performance among international students.

Based on the study findings, universities in Jordan ought to provide exercise breaks, improve wellness programs, and promote campaigns related to physical activities, such as activities that reduce stress and improve mental health. This will positively impact the academic performance of students, especially for non-Jordanian students who are suffering from adaptation challenges.

## Conclusion

Overall, the findings of this research emphasize that medical students engage in moderate levels of physical activity. A lack of time and energy were significant barriers in our study sample, while health maintenance and tension relief emerged as the primary motivators. Potential sociocultural and institutional influences were suggested by the significant variation in academic performance based on age, university, and nationality in our study sample. The need for targeted strategies to encourage regular physical activity and support academic achievement across a variety of student groups is suggested by these findings.

## References

[1] Khasawneh W, Obeidat N, Albiss B, El-Salem K. Selection criteria and match results for postgraduate residency programs: A cross-sectional model from a major academic center in Jordan. Ann Med Surg (Lond). 2020;59:199–203. doi: 10.1016/j.amsu.2020.10.001 33204413 PMC7647935

[2] McManus IC, Smithers E, Partridge P, Keeling A, Fleming PR. A levels and intelligence as predictors of medical careers in UK doctors: 20 year prospective study. BMJ. 2003;327(7407):139–42. doi: 10.1136/bmj.327.7407.139 12869457 PMC165701

[3] Marwan Y, Ayed A. Selection criteria of residents for residency programs in Kuwait. BMC Med Educ. 2013;13:4. doi: 10.1186/1472-6920-13-4 23331670 PMC3552967

[4] Abadi B, Zamani GH. Factors affecting agricultural students’ academic achievement: a case of college of agriculture, Shiraz University. 2010;

[5] Mega C, Ronconi L, De Beni R. What makes a good student? How emotions, self-regulated learning, and motivation contribute to academic achievement. Journal of Educational Psychology. 2014;106(1):121–31. doi: 10.1037/a0033546

[6] Hayden LJ, Jeong SY, Norton CA. An Analysis of Factors Affecting Mature Age Students’ Academic Success in Undergraduate Nursing Programs: A Critical Literature Review. International Journal of Nursing Education Scholarship. 2016;13(1):127–38. doi: 10.1515/ijnes-2015-0086 27906668

[7] Bye D, Pushkar D, Conway M. Motivation, Interest, and Positive Affect in Traditional and Nontraditional Undergraduate Students. Adult Education Quarterly. 2007;57(2):141–58. doi: 10.1177/0741713606294235

[8] Gomes da Silva S, Arida RM. Physical activity and brain development. Expert Rev Neurother. 2015;15(9):1041–51. doi: 10.1586/14737175.2015.1077115 26289488

[9] Donnelly JE, Hillman CH, Castelli D, Etnier JL, Lee S, Tomporowski P, et al. Physical Activity, Fitness, Cognitive Function, and Academic Achievement in Children: A Systematic Review. Med Sci Sports Exerc. 2016;48(6):1197–222. doi: 10.1249/MSS.0000000000000901 27182986 PMC4874515

[10] Loprinzi PD, Cardinal BJ, Loprinzi KL, Lee H. Benefits and environmental determinants of physical activity in children and adolescents. Obes Facts. 2012;5(4):597–610. doi: 10.1159/000342684 22986648

[11] Tomporowski PD, Davis CL, Miller PH, Naglieri JA. Exercise and Children’s Intelligence, Cognition, and Academic Achievement. Educ Psychol Rev. 2008;20(2):111–31. doi: 10.1007/s10648-007-9057-0 19777141 PMC2748863

[12] Haskell WL, Lee I-M, Pate RR, Powell KE, Blair SN, Franklin BA, et al. Physical activity and public health: updated recommendation for adults from the American College of Sports Medicine and the American Heart Association. Circulation. 2007;116(9):1081–93. doi: 10.1161/CIRCULATIONAHA.107.185649 17671237

[13] Sanchis-Gomar F, Quilis CP, Lucia A. Antidepressant Effects of Exercise: A Role for the Adiponectin-PGC-1α-kynurenine Triad? J Cell Physiol. 2015;230(10):2328–9. doi: 10.1002/jcp.24964 25739976

[14] Álvarez-Bueno C, Pesce C, Cavero-Redondo I, Sánchez-López M, Garrido-Miguel M, Martínez-Vizcaíno V. Academic Achievement and Physical Activity: A Meta-analysis. Pediatrics. 2017;140(6):e20171498. doi: 10.1542/peds.2017-1498 29175972

[15] Donnelly JE, Hillman CH, Castelli D, Etnier JL, Lee S, Tomporowski P, et al. Physical Activity, Fitness, Cognitive Function, and Academic Achievement in Children: A Systematic Review. Med Sci Sports Exerc. 2016;48(6):1197–222. doi: 10.1249/MSS.0000000000000901 27182986 PMC4874515

[16] Abdel-Salam D, Abdel-Khalek E. Pattern and Barriers of Physical Activity among Medical Students of Al-Jouf University, Saudi Arabia. Journal of High Institute of Public Health. 2016;46(2):41–8. doi: 10.21608/jhiph.2016.20080

[17] Ghassab-Abdollahi N, Shakouri SK, Aghdam AT, Farshbaf-Khalili A, Abdolalipour S, Farshbaf-Khalili A. Association of quality of life with physical activity, depression, and demographic characteristics and its predictors among medical students. J Educ Health Promot. 2020;9:147. doi: 10.4103/jehp.jehp_91_20 32766332 PMC7377133

[18] Wattanapisit A, Fungthongcharoen K, Saengow U, Vijitpongjinda S. Physical activity among medical students in Southern Thailand: a mixed methods study. BMJ Open. 2016;6(9):e013479. doi: 10.1136/bmjopen-2016-013479 27678548 PMC5051498

[19] Janampa-Apaza A, Pérez-Mori T, Benites L, Meza K, Santos-Paucar J, Gaby-Pérez R, et al. Physical activity and sedentary behavior in medical students at a Peruvian public university. Medwave. 2021;21(5):e8210. doi: 10.5867/medwave.2021.05.8210 34214068

[20] Alhemedi AJ, Qasaimeh MG, Abdo N, Elsalem L, Qaadan D, Alomari E, et al. Depression Among University Students in Jordan After the COVID-19 Pandemic: A Cross-Sectional Study. Psychol Res Behav Manag. 2023;16:4237–49. doi: 10.2147/PRBM.S436293 37873060 PMC10590589

[21] Sajwani RA, Shoukat S, Raza R, Shiekh MM, Rashid Q, Siddique MS, et al. Knowledge and practice of healthy lifestyle and dietary habits in medical and non-medical students of Karachi, Pakistan. J Pak Med Assoc. 2009;59(9):650–5. 19750870

[22] Deisz M, Papproth C, Ambler E, Glick M, Eno C. Correlates and Barriers of Exercise, Stress, and Wellness in Medical Students. Med Sci Educ. 2024;34(6):1433–44. doi: 10.1007/s40670-024-02134-5 39758502 PMC11699034

[23] Satti MZ, Khan TM, Qurat-Ul-Ain Q-U-A, Azhar MJ, Javed H, Yaseen M, et al. Association of Physical Activity and Sleep Quality with Academic Performance Among Fourth-year MBBS Students of Rawalpindi Medical University. Cureus. 2019;11(7):e5086. doi: 10.7759/cureus.5086 31516795 PMC6721912

[24] Naser AY, Dahmash EZ, Al‐Rousan R, Alwafi H, Alrawashdeh HM, Ghoul I, et al. Mental health status of the general population, healthcare professionals, and university students during 2019 coronavirus disease outbreak in Jordan: A cross‐sectional study. Brain and Behavior. 2020;10(8). doi: 10.1002/brb3.1730 PMC7361060 32578943

[25] Abuhamdah SMA, Naser AY. Smart phone addiction and its mental health risks among university students in Jordan: a cross-sectional study. BMC Psychiatry. 2023;23(1):812. doi: 10.1186/s12888-023-05322-6 37936164 PMC10631016

[26] Abuhamdah SMA, Naser AY, Abdelwahab GM, AlQatawneh A. The Prevalence of Mental Distress and Social Support among University Students in Jordan: A Cross-Sectional Study. Int J Environ Res Public Health. 2021;18(21):11622. doi: 10.3390/ijerph182111622 34770136 PMC8583308

[27] Naser AY, Alwafi H, Itani R, Alzayani S, Qadus S, Al-Rousan R, et al. Nomophobia among university students in five Arab countries in the Middle East: prevalence and risk factors. BMC Psychiatry. 2023;23(1):541. doi: 10.1186/s12888-023-05049-4 37496010 PMC10369834

[28] Booth FW, Roberts CK, Laye MJ. Lack of exercise is a major cause of chronic diseases. Compr Physiol. 2012;2(2):1143–211. doi: 10.1002/cphy.c110025 23798298 PMC4241367

[29] Stanford FC, Durkin MW, Stallworth JR, Powell CK, Poston MB, Blair SN. Factors that influence physicians’ and medical students’ confidence in counseling patients about physical activity. J Prim Prev. 2014;35(3):193–201. doi: 10.1007/s10935-014-0345-4 24682887 PMC6820677

[30] Altaim TA, Sundaram Subramanian S, Sam A, Alenezi L, Ramanathan K, Mustafa Gaowgzeh RA, et al. The relationship between physical activity and academic performance among health professional students: a correlational study. Retos. 2025;66:872–81. doi: 10.47197/retos.v66.113759

[31] Chung Q-E, Abdulrahman SA, Khan MKJ, Sathik HBJ, Rashid A. The Relationship between Levels of Physical Activity and Academic Achievement among Medical and Health Sciences Students at Cyberjaya University College of Medical Sciences. Malays J Med Sci. 2018;25(5):88–102. doi: 10.21315/mjms2018.25.5.9 30914866 PMC6419888

[32] Dyrbye LN, Satele D, Shanafelt TD. Healthy Exercise Habits Are Associated With Lower Risk of Burnout and Higher Quality of Life Among U.S. Medical Students. Acad Med. 2017;92(7):1006–11. doi: 10.1097/ACM.0000000000001540 28030419

[33] Fisher JJ, Kaitelidou D, Samoutis G. Happiness and physical activity levels of first year medical students studying in Cyprus: a cross-sectional survey. BMC Med Educ. 2019;19(1):475. doi: 10.1186/s12909-019-1790-9 31888602 PMC6937866

[34] Taylor CE, Scott EJ, Owen K. Physical activity, burnout and quality of life in medical students: A systematic review. Clin Teach. 2022;19(6):e13525. doi: 10.1111/tct.13525 36052814 PMC9826463

[35] Sánchez-Miguel PA, Leo FM, Amado D, Pulido JJ, Sánchez-Oliva D. Relationships between Physical Activity Levels, Self-Identity, Body Dissatisfaction and Motivation among Spanish High School Students. J Hum Kinet. 2017;59:29–38. doi: 10.1515/hukin-2017-0145 29134046 PMC5680684

[36] Akhmad I, Heri Z, Hariadi H. Physical activity levels among Malaysian university and state university of Medan students: gender difference and the influence of BMI. Retos: nuevas tendencias en educación física, deporte y recreación. 2024;60:429–38.

[37] Singh A, Uijtdewilligen L, Twisk JWR, van Mechelen W, Chinapaw MJM. Physical activity and performance at school: a systematic review of the literature including a methodological quality assessment. Arch Pediatr Adolesc Med. 2012;166(1):49–55. doi: 10.1001/archpediatrics.2011.716 22213750

[38] Slade AN, Kies SM. The relationship between academic performance and recreation use among first-year medical students. Med Educ Online. 2015;20:25105. doi: 10.3402/meo.v20.25105 25819693 PMC4376935

[39] McIsaac J-LD, Kirk SFL, Kuhle S. The Association between Health Behaviours and Academic Performance in Canadian Elementary School Students: A Cross-Sectional Study. Int J Environ Res Public Health. 2015;12(11):14857–71. doi: 10.3390/ijerph121114857 26610537 PMC4661684

[40] Eldridge JA, Palmer TB, Gillis K, Lloyd R, Squires WG Jr, Murray TD. Comparison of Academic and Behavioral Performance between Athletes and Non-athletes. Int J Exerc Sci. 2014;7(1):3–13. doi: 10.70252/UVXP4768 27182397 PMC4831893

[41] Bass RW, Brown DD, Laurson KR, Coleman MM. Physical fitness and academic performance in middle school students. Acta Paediatr. 2013;102(8):832–7. doi: 10.1111/apa.12278 23621404

[42] Al-Drees A, Abdulghani H, Irshad M, Baqays AA, Al-Zhrani AA, Alshammari SA, et al. Physical activity and academic achievement among the medical students: A cross-sectional study. Med Teach. 2016;38 Suppl 1:S66-72. doi: 10.3109/0142159X.2016.1142516 26984037

[43] Miller KE, Melnick MJ, Barnes GM, Farrell MP, Sabo D. Untangling the Links among Athletic Involvement, Gender, Race, and Adolescent Academic Outcomes. Sociol Sport J. 2005;22(2):178–93. doi: 10.1123/ssj.22.2.178 16467902 PMC1343519

[44] Meacham J. Are Physically Active College Students More Successful Academically Than Their Inactive Peers? University of New Orleans; 2015.

[45] Martin A, Saunders DH, Shenkin SD, Sproule J. Lifestyle intervention for improving school achievement in overweight or obese children and adolescents. Cochrane Database Syst Rev. 2014;(3):CD009728. doi: 10.1002/14651858.CD009728.pub2 24627300

[46] Sabet F, Zoghoul S, Alahmad M, Al Qudah H. The influence of gender on clinical examination skills of medical students in Jordan: a cross-sectional study. BMC Med Educ. 2020;20(1). doi: 10.1186/s12909-020-02002-x PMC7110726 32234037

[47] Aljayyousi GF, Abu Munshar M, Al-Salim F, Osman ER. Addressing context to understand physical activity among Muslim university students: the role of gender, family, and culture. BMC Public Health. 2019;19(1). doi: 10.1186/s12889-019-7670-8 PMC6829810 31690307

[48] Dąbrowska-Galas M, Ptaszkowski K, Dąbrowska J. Physical Activity Level, Insomnia and Related Impact in Medical Students in Poland. Int J Environ Res Public Health. 2021;18(6):3081. doi: 10.3390/ijerph18063081 33802730 PMC8002503

[49] Schlickmann DW, Kock KdS. Level of Physical Activity Knowledge of Medical Students in a Brazilian University. J Lifestyle Med. 2022;12(1):47–55. doi: 10.15280/jlm.2022.12.1.47 35300037 PMC8918374

[50] Laishram JV, Akoijam B. Medical students’ practice, attitudes, and motives toward physical activity: A cross-sectional study. J Med Soc. 2020;34(3):144. doi: 10.4103/jms.jms_1_21

[51] Rao CR, Darshan B, Das N, Rajan V, Bhogun M, Gupta A. Practice of Physical Activity among Future Doctors: A Cross Sectional Analysis. Int J Prev Med. 2012;3(5):365–9. 22708033 PMC3372079

[52] Abdelrahman MA, Abuelgasim A, Hassan Marouf HA, Alalim AM. Physical Activity and Academic Achievement among Omdurman Islamic University Medical Students 2021. medRxiv. 2022:2022.12.12.22283383. doi: 10.1101/2022.12.12.22283383

[53] Macilwraith P, Bennett D. Burnout and Physical Activity in Medical Students. Ir Med J. 2018;111(3):707. 30376225

[54] Bogdanavičienė K, Gudavičiūtė G, Alšauskė SV. Effect of physical activity on academic achievement of medical students. Medical Sciences. 2022.

[55] Padmapriya K, Krishna P, Rasu T. Prevalence and patterns of physical activity among medical students in Bangalore, India. Electron Physician. 2013;5(1):606–10. doi: 10.14661/2013.606-610 26120390 PMC4477776

[56] Pitsavos C, Panagiotakos DB, Lentzas Y, Stefanadis C. Epidemiology of leisure-time physical activity in socio-demographic, lifestyle and psychological characteristics of men and women in Greece: the ATTICA Study. BMC Public Health. 2005;5:37. doi: 10.1186/1471-2458-5-37 15836794 PMC1087851

[57] WHO. Physical activity. 5/2, 2025. https://www.who.int/news-room/fact-sheets/detail/physical-activity

[58] Likus W, Milka D, Bajor G, Jachacz-Łopata M, Dorzak B. Dietary habits and physical activity in students from the Medical University of Silesia in Poland. Rocz Panstw Zakl Hig. 2013;64(4):317–24. 24693717

[59] Ilić M, Pang H, Vlaški T, Grujičić M, Novaković B. Motives and Barriers for Regular Physical Activity among Medical Students from the Western Balkans (South-East Europe Region). Int J Environ Res Public Health. 2022;19(23):16240. doi: 10.3390/ijerph192316240 36498317 PMC9736948

[60] Connelly M, Waites S, Kaeley D, Brewster P, Sun Z, Randall Worth. Fit for Health? Levels of Physical Activity Among Preclinical and Clinical Medical Students. Am J Lifestyle Med. 2023;17(6):813–30. doi: 10.1177/15598276231161501 38511116 PMC10948927

[61] Alecu S, Onea GA, Badau D. The Relationship Between Motivation for Physical Activity, Physical Activity Level, and Body Mass Index for University Students. Sports (Basel). 2025;13(4):96. doi: 10.3390/sports13040096 40278722 PMC12031277

[62] Alnofaiey YH, Atallah HM, Alrawqi MK, Alghamdi H, Almalki MG, Almaleky JS, et al. Correlation of Physical Activity to Mental Health State and Grade Point Average Among Medical Students in Saudi Arabia: A Cross-Sectional Study. Cureus. 2023;15(6):e40253. doi: 10.7759/cureus.40253 37440798 PMC10335324

[63] Guerrero-López JB, Monterrosas AM, Reyes-Carmona C, Arrioja Guerrero A, Navarrete-Martínez A, Flores Morones F, et al. Factors related to academic performance in medical students. Salud Ment. 2023;46(4):193–200. doi: 10.17711/sm.0185-3325.2023.024

[64] Egwuatu VE, Umeora OUJ. A comparative study of marital status on the academic performance of the female medical undergraduate in a Nigerian university. Niger Postgrad Med J. 2007;14(3):175–9. doi: 10.4103/1117-1936.177540 17767198

[65] Nto N, Obikili E, Anyanwu G, Agu A, Esom E, Ezugworie J. Effect of age, premedical academic performance, and entry bias on students’ performance in final preclinical examination at the University of Nigeria Medical School. J Exp Clin Anat. 2019;18(1):6. doi: 10.4103/jeca.jeca_2_19

[66] Salahdeen H, Murtala B. Relationship between admission grades and performances of students in the first professional examination in a new medical school. African Journal of Biomedical Research. 2006;8(1). doi: 10.4314/ajbr.v8i1.35760

[67] Olaleye SB, Salami HA. Predictors of academic performance in the pre-clinical sciences: effects of age, sex and mode of admission at the Maiduguri Medical School. Afr J Med Med Sci. 1997;26(3–4):189–90. 10456169

[68] Egwu OA, Anyanwu GE. Five-year survey of medical student attrition in a medical school in Nigeria: a pilot study. Adv Med Educ Pract. 2010;1:53–7. doi: 10.2147/amep.S13395 23745063 PMC3643137

[69] Niemi PM, Vainiomäki PT. Medical students’ distress--quality, continuity and gender differences during a six-year medical programme. Med Teach. 2006;28(2):136–41. doi: 10.1080/01421590600607088 16707294

[70] Kötter T, Wagner J, Brüheim L, Voltmer E. Perceived Medical School stress of undergraduate medical students predicts academic performance: an observational study. BMC Med Educ. 2017;17(1):256. doi: 10.1186/s12909-017-1091-0 29246231 PMC5732510

[71] Bramness JG, Fixdal TC, Vaglum P. Effect of medical school stress on the mental health of medical students in early and late clinical curriculum. Acta Psychiatr Scand. 1991;84(4):340–5. doi: 10.1111/j.1600-0447.1991.tb03157.x 1746285

[72] Mosley TH Jr, Perrin SG, Neral SM, Dubbert PM, Grothues CA, Pinto BM. Stress, coping, and well-being among third-year medical students. Acad Med. 1994;69(9):765–7. doi: 10.1097/00001888-199409000-00024 8074778

[73] Park CL, Adler NE. Coping style as a predictor of health and well-being across the first year of medical school. Health Psychol. 2003;22(6):627–31. doi: 10.1037/0278-6133.22.6.627 14640860

[74] Moffat KJ, McConnachie A, Ross S, Morrison JM. First year medical student stress and coping in a problem-based learning medical curriculum. Med Educ. 2004;38(5):482–91. doi: 10.1046/j.1365-2929.2004.01814.x 15107082

[75] Dyrbye LN, Thomas MR, Shanafelt TD. Medical student distress: causes, consequences, and proposed solutions. Mayo Clin Proc. 2005;80(12):1613–22. doi: 10.4065/80.12.1613 16342655

[76] Ferguson E, James D, Madeley L. Factors associated with success in medical school: systematic review of the literature. BMJ. 2002;324(7343):952–7. doi: 10.1136/bmj.324.7343.952 11964342 PMC102330

[77] Bitran M, Zúñiga D, Pedrals N, Padilla O, Mena B. Medical students’ change in learning styles during the course of the undergraduate program: from “thinking and watching” to “thinking and doing”. Can Med Educ J. 2012;3(2):e86-97. doi: 10.36834/cmej.36587 26451190 PMC4563626

[78] Liu Y, Lyu D, Xie S, Yao Y, Liu J, Lu B, et al. The impact of grade point average on medical students’ perception of the learning environment: a multicenter cross-sectional study across 12 Chinese medical schools. BMC Med Educ. 2025;25(1):448. doi: 10.1186/s12909-025-06977-3 40148831 PMC11951772

[79] Al Alwan I, Al Kushi M, Tamim H, Magzoub M, Elzubeir M. Health sciences and medical college preadmission criteria and prediction of in-course academic performance: a longitudinal cohort study. Adv Health Sci Educ Theory Pract. 2013;18(3):427–38. doi: 10.1007/s10459-012-9380-1 22669557

[80] Kumwenda B, Cleland JA, Walker K, Lee AJ, Greatrix R. The relationship between school type and academic performance at medical school: a national, multi-cohort study. BMJ Open. 2017;7(8):e016291. doi: 10.1136/bmjopen-2017-016291 28860227 PMC5589012

[81] Van der Veken J, Valcke M, De Maeseneer J, Derese A. Impact of the transition from a conventional to an integrated contextual medical curriculum on students’ learning patterns: a longitudinal study. Med Teach. 2009;31(5):433–41. doi: 10.1080/01421590802141159 18825559

[82] Kim T, Chang J-Y, Myung SJ, Chang Y, Park KD, Park WB, et al. Predictors of Undergraduate and Postgraduate Clinical Performance: A Longitudinal Cohort Study. J Surg Educ. 2016;73(4):715–20. doi: 10.1016/j.jsurg.2016.03.006 27142720

[83] Kinika C, Egbo R. Exploring the impact of migration on the mental health of international students and its effects on academic performance in the UK. BMC Immunology. 2024.

[84] Henning MA, Krägeloh C, Moir F, Doherty I, Hawken SJ. Quality of life: international and domestic students studying medicine in New Zealand. Perspect Med Educ. 2012;1(3):129–42. doi: 10.1007/s40037-012-0019-y 23316469 PMC3540361

[85] Jiang Q, Horta H, Yuen M. High- and low-achieving international medical students’ perceptions of the factors influencing their academic performance at Chinese universities. Med Educ Online. 2024;29(1):2300194. doi: 10.1080/10872981.2023.2300194 38166562 PMC10769138

[86] Aleidi S, Elayah E, Zraiqat D, Abdallah R, AL-Iede M. Factors affecting the academic performance of medical, dental, and pharmacy students in Jordan. Jordan Journal of Pharmaceutical Sciences. 2020;13(2).

[87] Jieyi H, Kiu CC, Baojian X. How academic performance influences social integration: The moderation effect of cultural distance among Chinese cross-borderers. Brain Behav. 2022;12(10):e2759. doi: 10.1002/brb3.2759 36102099 PMC9575612

[88] Landoll RR, Bennion LD, Maggio LA. Understanding Excellence: a Qualitative Analysis of High-Performing Learner Study Strategies. Med Sci Educ. 2021;31(3):1101–8. doi: 10.1007/s40670-021-01279-x 34457953 PMC8368812

[89] Salih S, Fageehi M, Hakami S, Ateya E, Hakami M, Hakami H, et al. Academic Difficulties Among Medical Students at Jazan University: A Case-Control Study. Adv Med Educ Pract. 2021;12:723–9. doi: 10.2147/AMEP.S307554 34234611 PMC8254185

