# Supplementary material for: Physical exercise and academic performance among students in the medical field at Jordanian Universities
Source: PLoS One. 2026 Jan 22;21(1):e0341310. doi: 10.1371/journal.pone.0341310 (PMC12826517; doi:10.1371/journal.pone.0341310)
Supplement: Attachment — Submitted filename: Response to reviewer.pdf [file pone.0341310.s004.pdf]

Response to reviewer:

Thank you for submitting the revised version (R2) and your detailed response. The manuscript is substantially improved in clarity and structure. In particular, the study objective is now more explicit, the analytical strategy is better organized, and the reporting is generally more consistent. I also note that the previously observed inconsistency in the reported proportion for "moderate" physical activity appears to have been corrected and is now aligned across the abstract/text/tables. The sample size reporting is also more consistent.

Before the manuscript can be accepted, please address the following minor but important points:

Language and copyediting (required)

- Thank you for this comment, the currently submitted manuscript has undergone complete proofreading.

The revised manuscript still contains multiple obvious typographical/spacing issues (e.g., merged words, duplicated letters, and formatting artifacts).

- Thank you for this comment, the currently submitted manuscript has undergone complete proofreading.

Please perform a final professional proofreading pass and ensure consistent formatting throughout (headings, spacing, punctuation, abbreviations).

- Thank you for this comment, the currently submitted manuscript has undergone complete proofreading.

Consistency between the response letter and Methods

Your response includes a contradiction regarding whether any questionnaire items were "adapted" from previously validated instruments.

Please ensure the response letter is internally consistent and fully aligned with the final Methods text. If items were adapted, specify from which sources; if not, remove any wording that implies adaptation.

- Thank you for this comment. We have now removed any wording that implies adaptation, see the method section.

Avoid overstatement of representativeness

Given the convenience/online recruitment approach, please avoid wording that implies the sampling strategy “ensures” or “guarantees” representativeness.

- Thank you for this comment. We have now addressed this comment, see line 121.

Please revise such statements to more neutral language (e.g., recruitment across different time windows/academic periods to increase coverage), and acknowledge this appropriately as a limitation where relevant.

- Thank you for this comment. We have now addressed this comment, see lines 100-107.
