# Supplementary material for: Physical exercise and academic performance among students in the medical field at Jordanian Universities
Source: PLoS One. 2026 Jan 22;21(1):e0341310. doi: 10.1371/journal.pone.0341310 (PMC12826517; doi:10.1371/journal.pone.0341310)
Supplement: Attachment — Submitted filename: Response to reviewer.docx [file pone.0341310.s003.docx]

Response to reviewer:

Thanks, your revision was very thorough and your answers detailed. The paper has become a much better job. The primary objective and analytic focus is now well defined, the sampling frame and administration of survey is better outlined, multivariate logistic regression application is more evident and the data is released in an appropriate way in a public repository. As well, you have refocused the Discussion to put your key findings into the Jordanian context, such as the non-relation between physical activity and academic performance and gender difference in physical activity participation.

Prior to acceptance of the paper, I would wish you consider the following minor issues:
Harmonize the sample size throughout the manuscript.
In several places the total sample is reported as 1,209, while one sentence still reads “1,290 209 university students”, which appears to be a residual editing artifact. Please ensure that the same total N is used consistently in the Abstract, Methods, Results, tables, and cover letter.

- Response: We have now addressed this comment.

Thorough language and style check.
The English is generally understandable, but several typographical and grammatical errors remain and should be corrected (e.g. “psychical activity” → “physical activity”, “Health improvement and stress relief their drove motivation” → “Health improvement and stress relief were the main motivators”, “factosfactors” → “factors”, “was utilized” → “were utilized”, consistent use of “cGPA/CGPA”, etc.). A careful proof-reading or professional language editing is recommended to meet journal standards.

- Response: We have now addressed this comment and conducted full proofreading for the manuscript.

Clarify questionnaire development.
In your response letter you state that some items were adapted from validated instruments but also that “No items were adapted from previous literature”, which is contradictory. Please make the Methods section explicit and internally consistent: either
(a) specify which items were adapted and cite the original sources, or
(b) state clearly that all items were developed de novo for this study based on the literature, and remove any mention of adaptation.

- Response: We have now clarified that we developed an anonymous questionnaire to examine the association between physical activity and academic performance among medical students in Jordan based on previous literature review.

Minor clarifications.

Explicitly state in the Methods that the sports-injury question referred to lifetime history (ever having had a sports-related injury), to match the explanation given in your response.

- Response: We have now addressed this comment, see lines 133-134.

Double-check all tables (especially Tables 2–4) for formatting, consistent decimal places, and p-value notation (e.g. use “p < 0.001” rather than “0.000”).

- Response: We have now addressed this comment.
